# Analysis of Environmental Effects on Leaf Temperature under Sunlight, High Pressure Sodium and Light Emitting Diodes

**DOI:** 10.1371/journal.pone.0138930

**Published:** 2015-10-08

**Authors:** Jacob A. Nelson, Bruce Bugbee

**Affiliations:** Crop Physiology Laboratory, Department of Plant Soils and Climate, Utah State University, Logan, Utah, United States of America; University of Western Sydney, AUSTRALIA

## Abstract

The use of LED technology is commonly assumed to result in significantly cooler leaf temperatures than high pressure sodium technology. To evaluate the magnitude of this effect, we measured radiation incident to and absorbed by a leaf under four radiation sources: clear sky sunlight in the field, sunlight in a glass greenhouse, and indoor plants under either high pressure sodium or light emitting diodes. We then applied a common mechanistic energy-balance model to compare leaf to air temperature difference among the radiation sources and environments. At equal photosynthetic photon flux, our results indicate that the effect of plant water status and leaf evaporative cooling is much larger than the effect of radiation source. If plants are not water stressed, leaves in all four radiation sources were typically within 2°C of air temperature. Under clear sky conditions, cool sky temperatures mean that leaves in the field are always cooler than greenhouse or indoor plants-when photosynthetic photon flux, stomatal conductance, wind speed, vapor pressure deficit, and leaf size are equivalent. As water stress increases and cooling via transpiration decreases, leaf temperatures can increase well above air temperature. In a near-worst case scenario of water stress and low wind, our model indicates that leaves would increase 6°, 8°, 10°, and 12°C above air temperature under field, LED, greenhouse, and HPS scenarios, respectively. Because LED fixtures emit much of their heat through convection rather than radiative cooling, they result in slightly cooler leaf temperatures than leaves in greenhouses and under HPS fixtures, but the effect of LED technology on leaf temperature is smaller than is often assumed. Quantifying the thermodynamic outputs of these lamps, and their physiological consequences, will allow both researchers and the horticulture industry to make informed decisions when employing these technologies.

## Introduction

Light emitting diodes (LEDs) have shown to be a disruptive technology in all areas of illumination. Large (>200 watt) LED fixtures have become commercially available for both sole source (growth chamber) and supplemental (greenhouse) plant lighting. At the same time, the more traditional high pressure sodium (HPS) technology has also improved in efficiency by as much as 60% [1]. Though the spectral effects of different light sources have been studied, there have been few studies on the thermodynamics of these new lighting systems. The thermal properties of fixtures are of consequence both for researchers, as differences in leaf temperature and transpiration can change experimental outcomes, and for the horticulture industry, as heating and cooling costs may be different under different sources and hot lamps may damage plant tissue.

The energy balance of leaves has long been studied in field conditions and a well-developed family of models is used to determine transpiration and leaf temperature over a wide range of environmental conditions, including controlled environments [2–5]. These models are well developed, and are used to predict values that are hard to measure directly, such as leaf temperature and evapotranspiration [6]. Models also provide the opportunity to compare individual parameters while keeping all other environmental conditions exactly the same. This facilitates comparison of radiation sources.

Although linearization of energy balance models, such as the Penman-Monteith equation, has been widely used, modern computing allows for more precise numerical solutions of leaf temperature. Widmoser [7] discusses the advantages of using numerical solutions.

A recent analysis showed that the conversion efficiency of electricity to photosynthetic photons of the most efficient commercial scale LED fixtures was equal to the most efficient HPS fixtures at 1.7 *μmol* photosynthetic photos per joule of electrical input [1]. They thus generate the same amount of thermal energy per photosynthetic photon. LED fixtures, however, dissipate much of their heat away from the plane they illuminate, while HPS fixtures dissipate more heat toward the plane they illuminate.

Elevated temperature reduces the lifespan of LEDs, so they are thermally-bonded to heat sinks where the thermal energy is removed by natural or fan-assisted convection and directed away from the plants they illuminate.

Conversely, HPS lamps operate at higher temperatures and thus generate more longwave radiation in the same direction as the photosynthetic radiation. This thermal radiation can be reduced using a barrier such as glass, but this reduces the photosynthetic radiation by about 10% (S1 Fig) and thus lowers the efficiency of the fixture.

The difference in how LED and HPS technologies dissipate thermal energy indicates that use of HPS fixtures will result in higher leaf temperatures. It is easy to misjudge the magnitude of this effect because HPS lamps are a far more concentrated light source than LEDs. Comparisons need to be made on the basis of equal photosynthetic photon flux (PPF).

Compared to sunlight and HPS lamps, LED fixtures emit almost no near infrared radiation (NIR; 700–3000 nm), but this radiation is not well absorbed by plant leaves. Photosynthetic (400 to 700 nm) and longwave (3,000 to 100,000 nm) radiation are about 95% absorbed, but non-photosynthetic solar NIR is only about 20% absorbed, and has a smaller effect on leaf heating. Unabsorbed radiation is either transmitted or reflected.

Our objective was to use a well-established energy-balance model to compare the leaf-to-air temperature difference in four radiation scenarios across multiple environments.

## Materials and Methods

### Radiation sources

We measured the radiation from four sources: clear sky sun in the field, clear sky sun in a greenhouse, and either HPS or LED fixtures in indoor environments (devoid of sunlight). The most efficient commercially-available HPS and LED fixtures (1.7 *μmol*/*J*; [1]) were used. The HPS fixture included a double-ended, 1000 W lamp (MASTER GreenPower, Philips Lighting, The Netherlands) in an efficient (less then 10% losses) luminaire (ePapillon, Lights Interaction, The Netherlands). The LED fixture was a 400 W, Red-Blue, passively cooled fixture (VividGro, Lighting Science Group, FL, USA). In order to quantify radiation conditions where the thermal and photosynthetic components are most intense, clear sky sun measurements were made near solar noon on a clear summer day in Logan, UT, USA. Greenhouse sun measurements were made under clear sky conditions in a typical glass greenhouse. All measurements were scaled to PPF to account for differences in fixture height. As a canopy gets closer to the fixture all types of radiation increase.

### Absorption of shortwave radiation

We measured shortwave absorption as the fraction of light that is neither transmitted nor reflected by a leaf.

**Fig 1:**
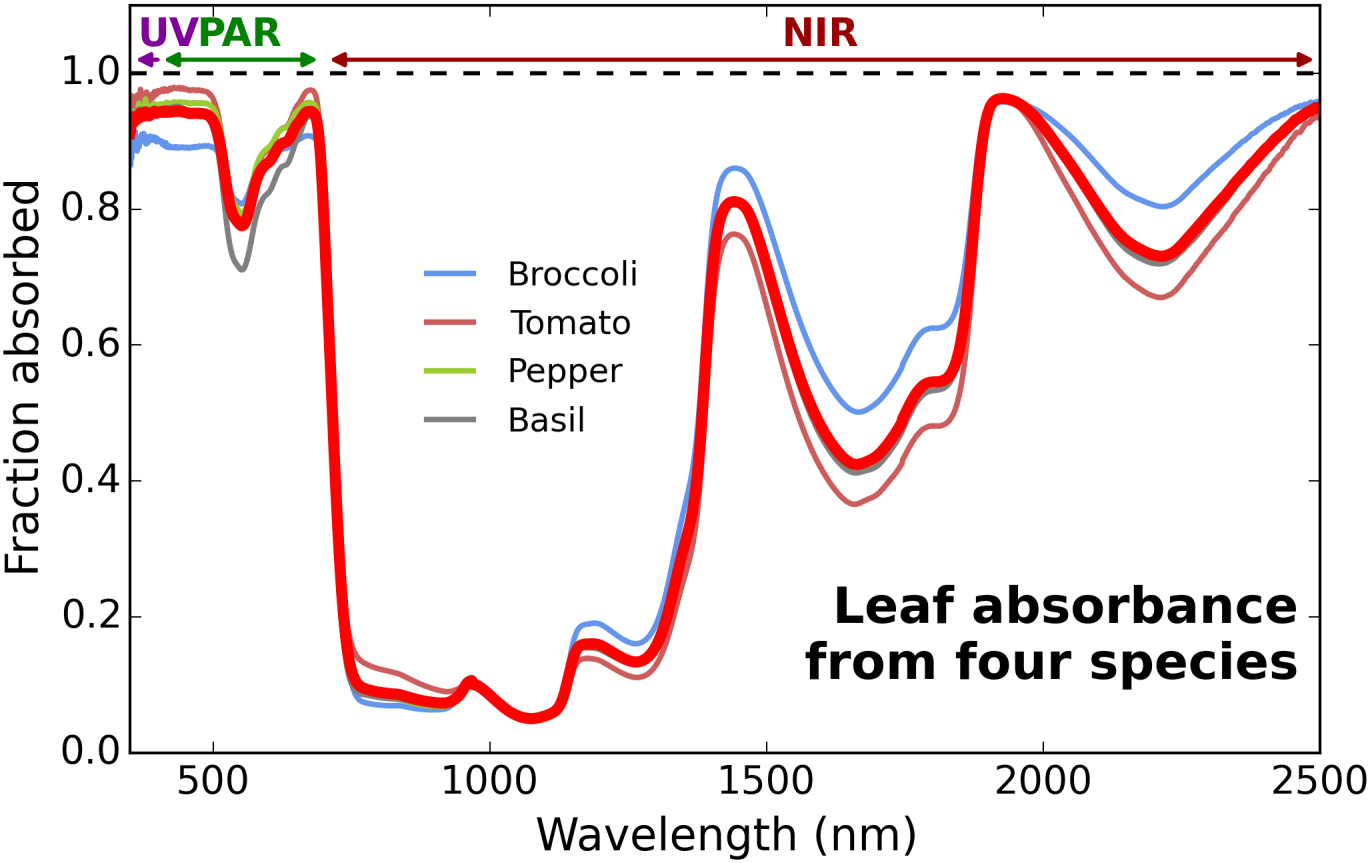
Average absorption (red line) of leaves from tomato, pepper, basil and broccoli. Variation among species is due to differences in leaf reflectance. The broccoli leaf had slightly higher reflectance of PAR than the other species. All plants were grown in a greenhouse.

Leaf absorption was determined by measurement of reflection and transmission between 350–2500 nm using a spectroradiometer (FieldSpec Pro, ASD Inc., Boulder, CO, USA) and a halogen light source. Transmission was measured through a single leaf at 90° from the leaf surface. Reflectance was made over a large black cavity with a small hole to mimic a black body, again at 90° from the leaf surface. Absorption was averaged among four species: tomato (S. lycopersicum), pepper (C. annuum), basil (O. basilicum), and broccoli (B. oleracea) (Fig 1) to incorporate a range of leaf types. Three separate leaves were measured on for each species. Average absorption was nearly identical to previously published values from multiple species and a variety of environments [8, 9]. The presented average absorption confirmed those of Noda et al. [10], which included leaves from 22 species.

**Fig 2:**
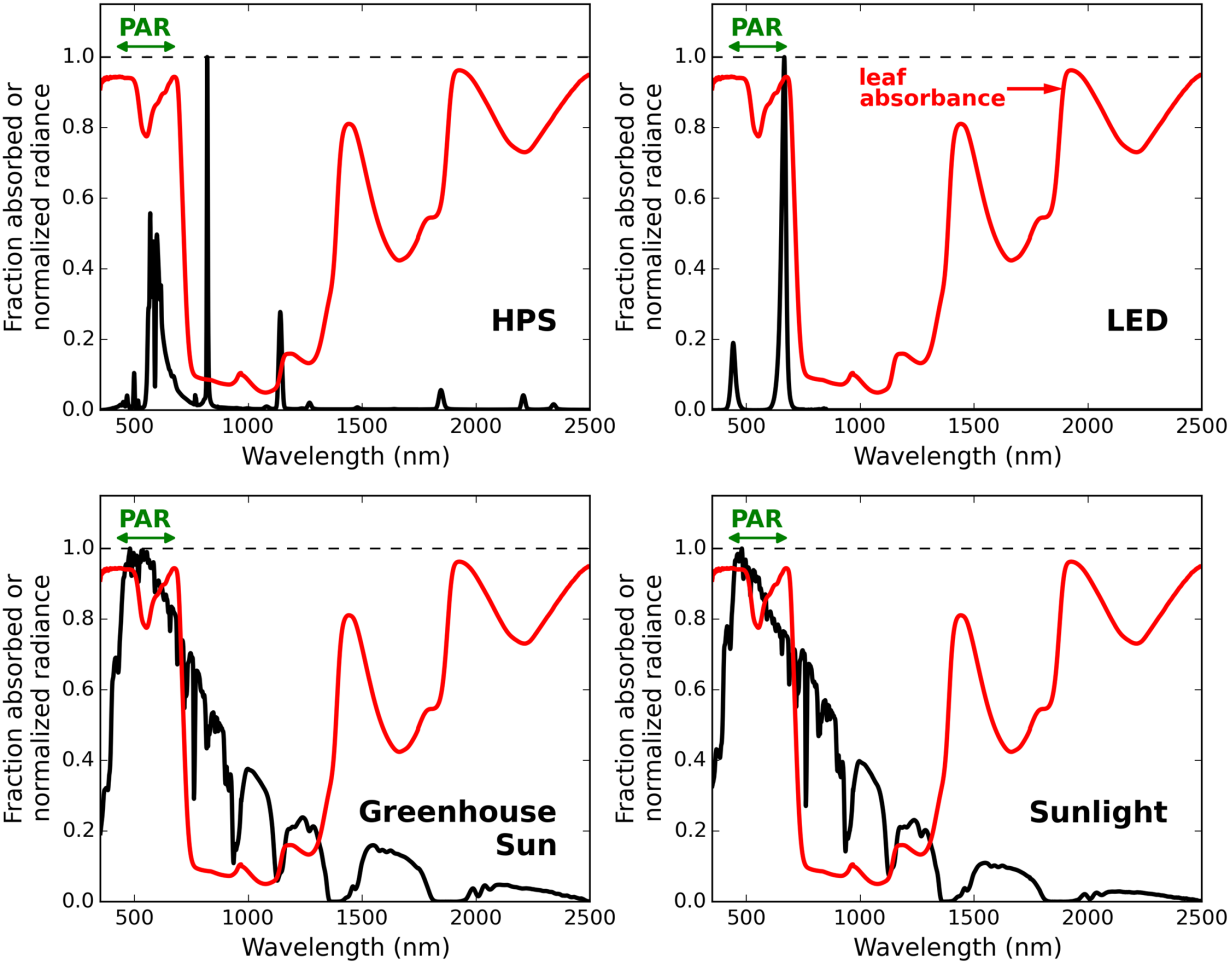
Radiance spectrum from four radiation sources (black line) and average leaf absorbance (red line). Electric lights (HPS and LED) output most of their radiation in the photosynthetic regions. Sunlight has significant NIR radiation, but this is poorly absorbed by leaves.

Relative spectral radiance of each radiation source was measured using the same spectroradiometer as above (Fig 2). Incoming shortwave (350–2,500 nm) and longwave (>3,000 nm) radiation measurements for each radiation scenario were made using a net radiometer (CNR1, Kipp & Zonen, the Netherlands). Photosynthetic photon flux (PPF; in moles per m^2^ per s) measurements were made using a recently calibrated quantum sensor (LI-190, LI-COR, Lincoln, NE, USA), and converted to photosynthetically active radiation (PAR; in watts per *m* ^2^) using spectral data for each light source and Planck’s equation (*E* = *hc*/*λ*). The absorbed radiation was normalized to equal incident PPF for each radiation source.

Because UV and photosynthetic radiation have much higher absorption than NIR, shortwave radiation was divided into three bands: ultraviolet (UV, 350–400 nm), PAR (400–700 nm), and near-infrared (NIR, 700–2500 nm). UV radiation below 350 nm is a minimal component from all radiation sources, and was not included in the analysis.

### Incoming and outgoing longwave radiation

Longwave radiation was separated into three components: sky longwave, source longwave, and emitted longwave. Sky longwave is the radiation emitted from either a clear sky (typically 300 *W*/*m* ^2^ or about -1°C), or the ceiling of the controlled environment (assumed to be 452 *W*/*m* ^2^ or about 28°C for all indoor cases). Source longwave is defined as the incoming longwave radiation from either the LED or HPS fixture, and was measured using a black body pyranometer (part of the net radiometer above). Incoming longwave radiation with the fixture present was subtracted from incoming longwave with the fixture absent. Source longwave was scaled with PPF. Emitted longwave is calculated using the Stefan-Boltzman law as outlined below. We assume the leaf is the same temperature as the surfaces below the leaf and thus there is no net longwave transfer.

### Energy balance model

We modeled a single top leaf because the uppermost leaves absorb about 75% of the incident radiation and have the greatest temperature differences.

Leaf temperature was calculated using the energy balance model that has been described, in detail, in both Campbell and Norman [11] and Monteith and Unsworth [12],

$$\begin{array}{c} R_{abs}=R_{emit}+C+\lambda E \end{array}$$ (1)

where,

$$\begin{array}{ccc} R_{abs} & = & \text{Absorbed}\;\text{radiation}\;\text{in}{\;}^{W}\;\;/{\;}_{m^{2}}\\ R_{emit} & = & \text{Emitted}\;\text{radiation}\;\text{via}\;\text{Stefan-Boltzmann}\;\text{law}\;\text{in}{\;}^{W}\;\;/{\;}_{m^{2}}\\ C & = & \text{Transfer}\;\text{of}\;\text{sensible}\;\text{heat}\;\text{via}\;\text{convection}\;\text{in}{\;}^{W}\;\;/{\;}_{m^{2}}\\ \lambda E & = & \text{Latent}\;\text{heat}\;\text{transfer}\;\text{in}{\;}^{W}\;\;/{\;}_{m^{2}} \end{array}$$

Assuming the system is at steady state, the absorbed radiation (*R*_*abs*_) must equal the sum of the emitted radiation (*R*_*emit*_), sensible (*C*) and latent (*λE*) heat transfer. Absorbed radiation was measured as described in the previous subsections. Emitted radiation is defined by the Stefan-Boltzmann law,

$$\begin{array}{c} R_{emit}=\varepsilon_{s}\sigma T_{L}^{4} \end{array}$$ (2)

where,

$$\begin{array}{ccc} \varepsilon_{s} & = & \text{Emissivity}\;\text{of}\;\text{the}\;\text{leaf}\;\text{(assumed}\;\text{to}\;\text{be}\;0.97)\\ \sigma & = & \text{The}\;\text{Stefan-Boltzmann}\;\text{constant}\;\text{or}\;5.67*10^{-8}{\;}^{W}\;\;/{\;}_{m^{2}K^{4}}\\ T_{L}^{4} & = & \text{Leaf}\;\text{temperature}\;\text{in}\;\text{Kelvin}\;\text{to}\;\text{the}\;\text{fourth}\;\text{power} \end{array}$$

The transfer of sensible heat (*C*), through convection, is defined as a function of the difference in leaf to air temperature and the boundary layer conductance such that,

$$\begin{array}{c} C=c_{p}g_{Ha}\left(T_{L}-T_{a}\right) \end{array}$$ (3)

where,

$$\begin{array}{ccc} c_{p} & = & \text{Specific}\;\text{heat}\;\text{of}\;\text{air}\;\text{at}\;\text{a}\;\text{constant}\;\text{pressure}\;\text{or}\;29.3{\;}^{J}\;\;/{\;}_{mol\;{\;}^{{}^{\circ}}\;C}\\ T_{L} & = & \text{Leaf}\;\text{temperature}\;\text{in}\;\text{Celsius}\\ T_{a} & = & \text{Air}\;\text{temperature}\;\text{in}\;\text{Celsius} \end{array}$$

Boundary layer conductance (*g*_*Ha*_ in ^*mol*^/_*m*^2^*s*_) is a semi-empirical function defined as,

$$\begin{array}{c} g_{Ha}=1.4*0.135\sqrt{\frac{u}{d}} \end{array}$$ (4)

where,

$$\begin{array}{ccc} 1.4 & = & \text{An}\;\text{empirical}\;\text{constant}\;\text{accounting}\;\text{for}\;\text{turbulance}\\ 0.135 & = & \text{An}\;\text{constant}\;\text{determined}\;\text{by}\;\text{the}\;\text{viscosity,}\;\text{density,}\;\text{and}\;\text{diffusivity}\;\text{of}\;\text{air}\\ u & = & \text{Wind}\;\text{speed}\;\text{in}\;{\;}^{m}\;\;/{\;}_{s}\\ d & = & \text{Characteristic}\;\text{dimension}\;\text{in}\;\text{meters}\;\text{or}\;\text{0.72}\;\text{times}\;\text{the}\;\text{maximum}\;\text{leaf}\;\text{width} \end{array}$$

Latent heat transfer (*λE*) is defined as a function of the vapor pressure deficit ($\frac{e_{s}\left(T_{L}\right)-e_{a}}{p_{a}}$) and the vapor conductance (*g*_*v*_ in ^*mol*^/_*m*^2^*s*_) such that,

$$\begin{array}{c} \begin{array}{c} \lambda E=\lambda g_{v}\frac{e_{s}(T_{L})-e_{a}}{p_{a}} \end{array} \end{array}$$ (5)

where,

$$\begin{array}{ccc} \lambda & = & \text{Latent}\;\text{heat}\;\text{of}\;\text{evaporation}\;\text{or}\;44{\;}^{kJ}\;\;/{\;}_{mol}\\ e_{s}(T_{L}) & = & \text{Saturation}\;\text{vapor}\;\text{pressure}\;\text{of}\;\text{water}\;\text{at}\;\text{leaf}\;\text{temperature}\;\text{in}\;\;kPa\\ e_{a} & = & \text{Partial}\;\text{pressure}\;\text{of}\;\text{water}\;\text{vapor}\;\text{in}\;\text{air}\;\text{in}\;\;kPa\\ p_{a} & = & \text{Atmospheric}\;\text{pressure}\;\text{or}\;101.3\;kPa \end{array}$$

Vapor conductance (*g*_*v*_) is a combination of both the vapor boundary (*g*_*va*_) and stomatal (*g*_*vs*_) conductances (both in c) such that,

$$\begin{array}{c} g_{v}=\frac{g_{vs}g_{va}}{g_{vs}+g_{va}} \end{array}$$ (6)

Stomatal conductance (*g*_*vs*_) typically varies between 0.1 ^*mol*^/_*m*^2^*s*_ for drought stressed plants and 0.5 ^*mol*^/_*m*^2^*s*_ for high transpiring plants. Vapor boundary conductance is defined similarly to Eq (4) with slightly different constants,

$$\begin{array}{c} g_{va}=1.4*0.147\sqrt{\frac{u}{d}} \end{array}$$ (7)

These components account for all significant energy paths. Other energy sources and sinks include photosynthesis and respiration, which are negligible in these conditions. Combining Eqs (1), (2), (3), and (5) gives a comprehensive overview of the model,

$$\begin{array}{c} R_{abs}=\varepsilon_{s}\sigma T_{L}^{4}+c_{p}g_{Ha}(T_{L}-T_{a})+\lambda g_{v}\frac{e_{s}(T_{L})-e_{a}}{p_{a}} \end{array}$$ (8)

The equation was solved for leaf temperature (*T*_*leaf*_) using an iterative approximation. Results are presented as the difference between leaf and air temperature (*T*_*leaf*_ − *T*_*air*_), as leaf temperature is only relevant in the context of it’s environment.

Some of the energy absorbed by leaves is used to fix CO_2_ into sucrose in the process of photosynthesis. The photosynthetic energy use in field conditions is typically less than 4% of the total absorbed energy and has thus been ignored in energy balance models. However, assuming optimal water and nitrogen, a moderate PPF and physiologically optimum CO_2_ enrichment, it is possible to increase the quantum yield of photosynthesis to 0.08 moles of CO_2_ fixed per mole of photons absorbed. Assuming respiration is 30% of photosynthesis, net metabolism can use about 8% of the absorbed shortwave energy [6]. This is still a small contribution to the total energy balance, and it would be similar for all radiation sources.

Code for the execution of the model can be found in supplemental information (S1 File).

### Sensitivity analysis

Excluding the radiation inputs, Eq (8) is ultimately a function of seven environmental variables: air temperature, relative humidity/vapor pressure deficit, wind speed, leaf size, sky temperature, stomatal conductance, and atmospheric pressure. Default values for each parameter were chosen to reflect typical growing conditions (as shown in figure captions).

**Fig 3:**
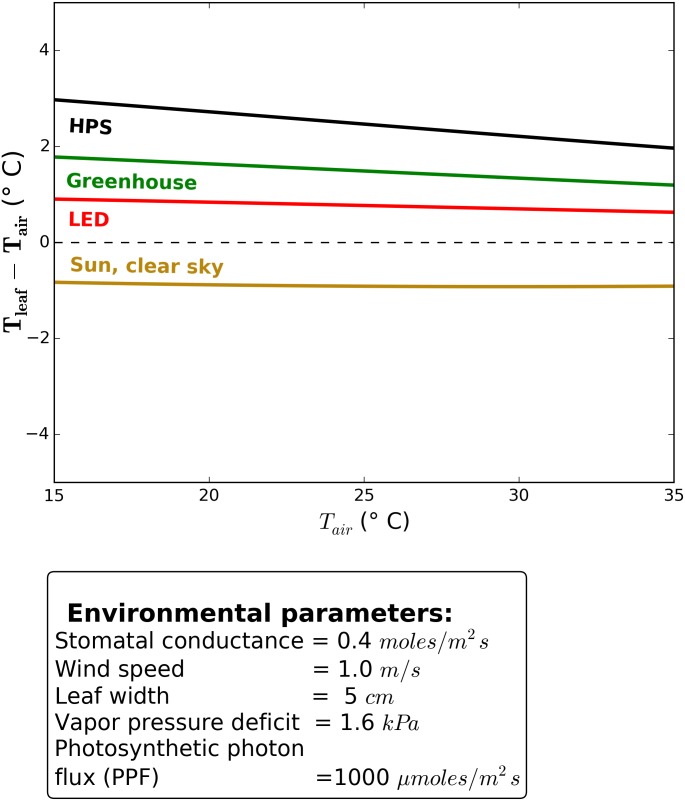
Leaf temperature response to air temperature. Vapor pressure deficit was held constant. Calculation is based on the average absoprtion of the four species measured. Differences among species were biologically insignificant.

Air temperature was held at 25°C, which is a common set point for greenhouses and growth chambers. Convective heat transfer from the lighting fixture and surrounding air is assumed to be controlled via the temperature control system before it would impact leaf temperature. When other environmental conditions are constant in the model, air temperature between 15° and 35°C has a minimal effect on leaf to air difference (Fig 3).

Environmental parameters were varied across a biologically significant range.

## Results and Discussion

The greatest variation among sources in incident radiation was in the near-infrared (NIR) and longwave bands (Table 1). NIR is poorly absorbed by leaves, so absorbed NIR was less than 30% of absorbed PAR energy for all sources.

**Table 1:** Incident radiation, fraction absorbed, and total absorbed radiation for each source. The absorbed radiation was normalized to a PPF of 1000 µmoles per m^2^ per s for each radiation source. This does not result in exactly equal PAR (in watts per m^2^) because of spectral differences among radiation sources. The total absorbed radiation for each source is shown in bold. Leaf temperature was held constant at 25°C. Net longwave exchange with lower leaves or surfaces was assumed to be zero.

	UV (350–400 nm)	PAR (400–700 nm)	NIR (700–2500 nm)	Source longwave	Sky longwave	Emitted longwave	Total
	Incident radiation (*W*/*m* ^2^)						
HPS	0.58	203	128	131	452	-448	467
LED	0.15	195	10	44	452	-448	253
Sun, greenhouse	18	219	252	0	452	-448	494
Sun, clear sky	19	219	289	0	300	-448	379
	Fraction absorbed						
HPS	0.939	0.870	0.263	0.97	0.97	0.97	0.71
LED	0.934	0.943	0.923	0.97	0.97	0.97	0.90
Sun, greenhouse	0.938	0.894	0.214	0.97	0.97	0.97	0.53
Sun, clear sky	0.937	0.894	0.207	0.97	0.97	0.97	0.33
	Total absorbed radiation (*W*/*m* ^2^)						
HPS	0.54	177	34	127	439	-435	**342**
LED	0.14	184	9	43	439	-435	**240**
Sun, greenhouse	17	196	54	0	439	-435	**271**
Sun, clear sky	18	196	60	0	291	-435	**130**

The indoor environments (LED, HPS, and greenhouse) had net positive longwave radiation, and the HPS fixture was significantly higher than the other sources. The effect of UV on absorbed radiation was less than 10% of absorbed PAR energy for all source.

### Effect of environment on leaf to air temperature difference

**Fig 4:**
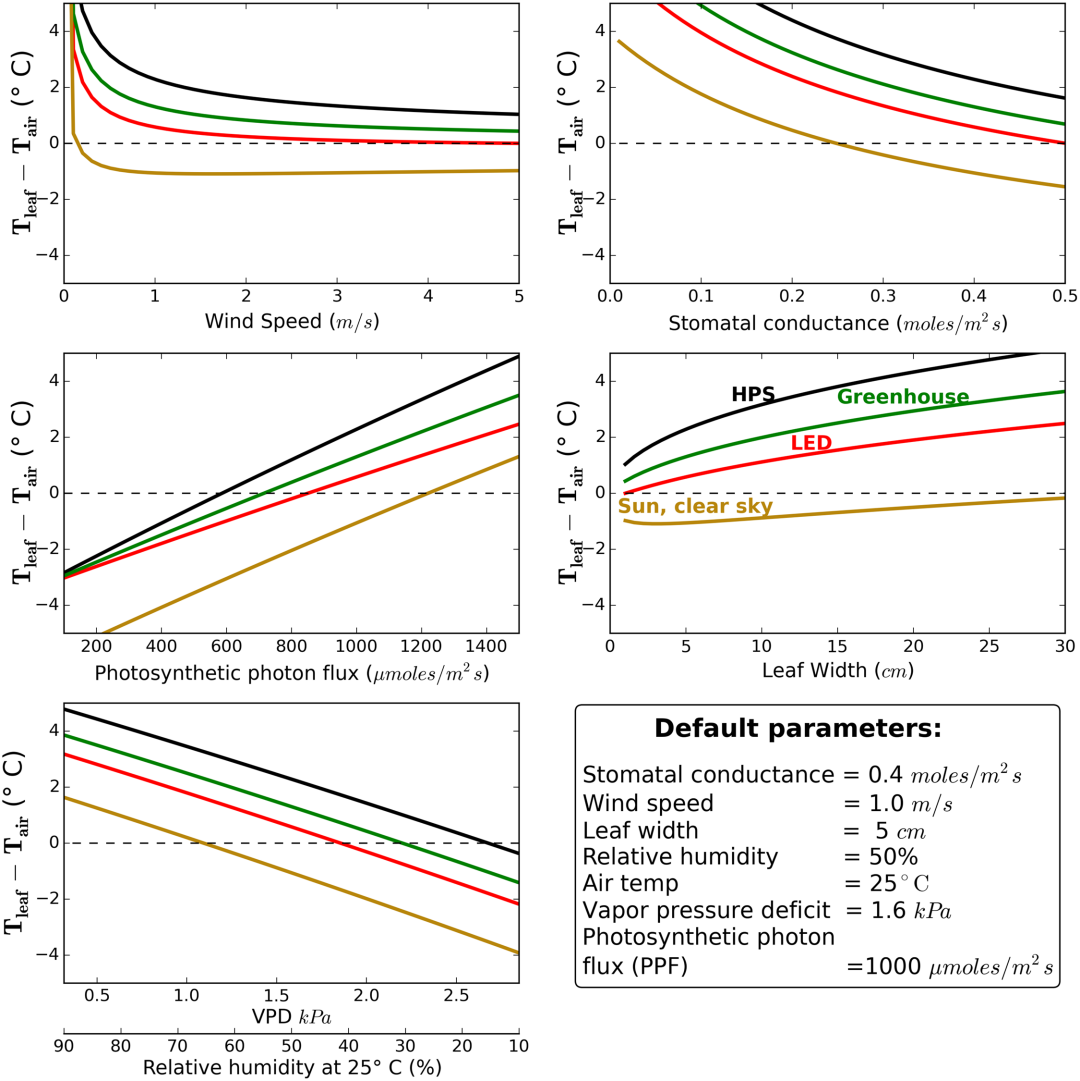
Calculated effects of environmental conditions on the difference between leaf temperature and air temperature under four radiation scenarios.

The leaf-to-air temperature difference, in all radiation scenarios, was less than 2°C except where parameters approached their extremes (Fig 4). The relative order did not change, regardless of environmental conditions, with HPS > greenhouse sun > LED > clear sky sunlight.

**Fig 5:**
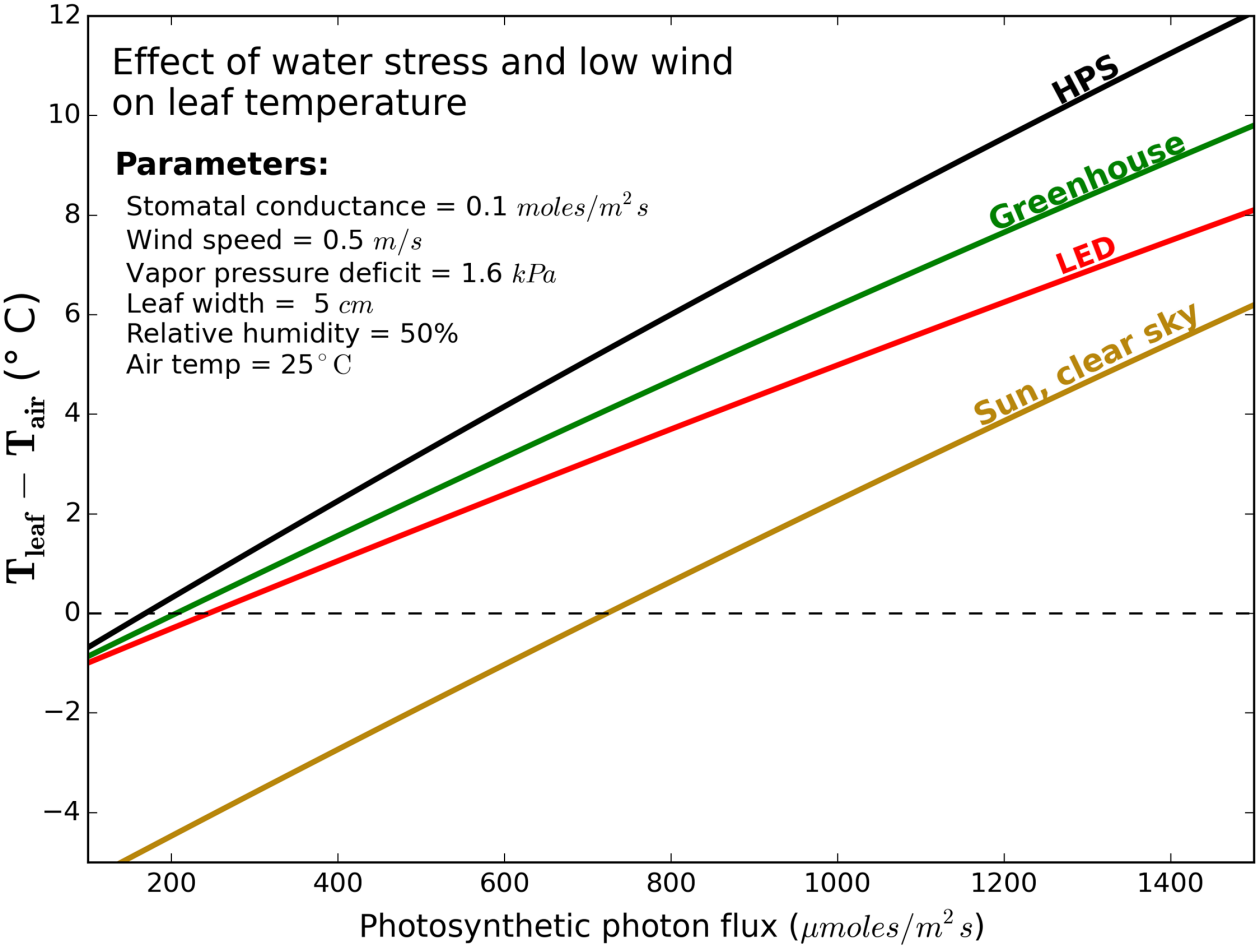
Calculated effects of PPF on the difference between leaf temperature and air temperature under four radiation scenarios in near worst-case conditions of water stress and low wind. Calculation is based on the average absoprtion of the four species measured. Differences among species was biologically insignificant.

Our modeled near worst-case conditions (water stress, high PPF, and low wind; Fig 5) increased the differences between lighting sources. The results indicate that leaf temperatures in near worst-case conditions can increase 6° to 12°C above air temperature depending on the radiation scenario.

### Differences in radiation absorption

There were significant differences among sources in the ratio of NIR to PPF, but NIR wavelengths are poorly absorbed by leaves (Table 1), thus the effect of NIR on leaf temperature is relatively small. Blanchard and Runkle [13] found leaf temperature to be 0.7° to 1.5°C lower under NIR reflective painted glass as opposed to neutral reflective painted glass with similar PPF conditions (about 1100 ^*μmole*^/_*m*^2^*s*_), though much of this difference was likely due to differences in air temperature, which was on average 0.8°C higher under neutral reflective paint. This further shows that though NIR is a significant source of energy, it’s impact on individual leaves is small.

Longwave radiation varied significantly among radiation sources and had the biggest effect on leaf temperature. Because incoming longwave radiation from clear sky conditions is significantly less than that from the ceiling of controlled environments, plants grown outdoors have lower absorbed net radiation. Even on overcast days, incoming long wave radiation in the field is typically lower than in a controlled environment.

Our analysis includes two of the most efficient fixtures available. Increases or decreases in efficiency will likely cause small differences in source longwave radiation, but the effect of changes in fixture efficiency would be relatively small compared to the effect of differences between the two technologies.

### Extrapolating from biophysical models to predict field performance

Simple measurements coupled with biophysical models are often used in large scale physiology experiments with confidence, such as using eddy covariance and satellite remote sensing to measure ecosystem latent heat flux and net ecosystem exchange. Building from well studied basic principles, simple measurements such as air temperature, leaf absorbance, and incoming and outgoing radiation, we can infer more complex relationships, such as transpiration and leaf temperature. Here we report the modeled relationships between environmental conditions, stomatal conductance, and leaf temperature.

### Effect of light source on transpiration

Increased leaf temperature causes increased transpiration. When incoming radiation and radiation capture by the crop are the same, the transpiration rate of crops in protected environments are thus higher than the same crops the field.

In the field, however, water loss by evaporation from the soil surface can make the combination of evaporation and transpiration higher than the combination of evaporation and transpiration in a controlled environment. If the effect of surface evaporation is removed and transpiration from only the leaves is considered, crops in a greenhouse would have a 35% higher transpiration rate than identical crops grown in the field, based on our model parameters.

Based on our presented model and the default parameters (Fig 4), the reduced leaf temperature under LED fixtures would decrease transpiration by 17% compared to HPS fixtures. This is a potentially significant reduction in transpiration, but differences in surface evaporation among cultural systems typically have a greater effect on crop water requirement than lamp type. For example, drip irrigation can decrease evaporation from surfaces and reduce the crop water requirement by 30 to 70%, in both greenhouses and in the field [14].

### Effect of elevated CO_2_

Controlled environments often add supplemental CO_2_, which can decrease stomatal conductance 10–40% [15, 16], and increase leaf temperature. The presented model indicates that a decrease in stomatal conductance of 30% in response to elevated CO_2_ would increase leaf temperature by 1°C in all radiation scenarios.

### Effect of light source on shoot tip temperature

Shoot tip temperature is often used to predict time to flower and plant development rates [17]. Our modeling approach is similar to that used by Shimizu et al. [5] and Faust and Heins [18] to predict shoot tip temperature, both of which found greater than 83% of their modeled values to be within 1°C of measured values. Because our models are similar, choice of lighting technology will likely affect shoot tip temperature, time to flower and plant development.

### Effect of light source on fruit and flower temperature

Our near-worst case analysis would likely be representative of flowers, fruits, and thick, dense plant parts that have low transpiration rates, including high value products such as tomatoes, strawberries, and *Cannabis* flowers. These thicker structures would absorb more radiation than a thin leaf. Our measurements show that while only 63% of HPS shortwave radiation is absorbed by the first leaf, a structure ten times thinker would absorb more than 80%. LED technology has the potential to reduce heating of these thick, low transpiring plant structures.

### Conclusions

The presented model indicates that the use of LED technology reduces leaf temperature by about 1.3°C compared to HPS technology under typical, indoor growing conditions. While this is a significant difference for some applications, the difference is smaller than the difference between indoor and outdoor leaves. Because of differences in net longwave radiation, a leaf in a controlled environment will be warmer than a leaf in the field under a clear sky, assuming equal PPF and similar environmental conditions. In conditions where leaves benefit from heating, such as a greenhouse in a cool climate, HPS technology more effectively transfers heat to canopies.

## Supporting Information

S1 Fig Transmission of radiation through a single pane of tempered glass. PAR was 89% transmitted.
(TIF)

S1 File Overview of code used to run the associated model. (PDF)
