# Supplementary figures and images for: Analysis of Environmental Effects on Leaf Temperature under Sunlight, High Pressure Sodium and Light Emitting Diodes
Source: PLoS One. 2015 Oct 8;10(10):e0138930. doi: 10.1371/journal.pone.0138930 (PMC4598144; doi:10.1371/journal.pone.0138930)

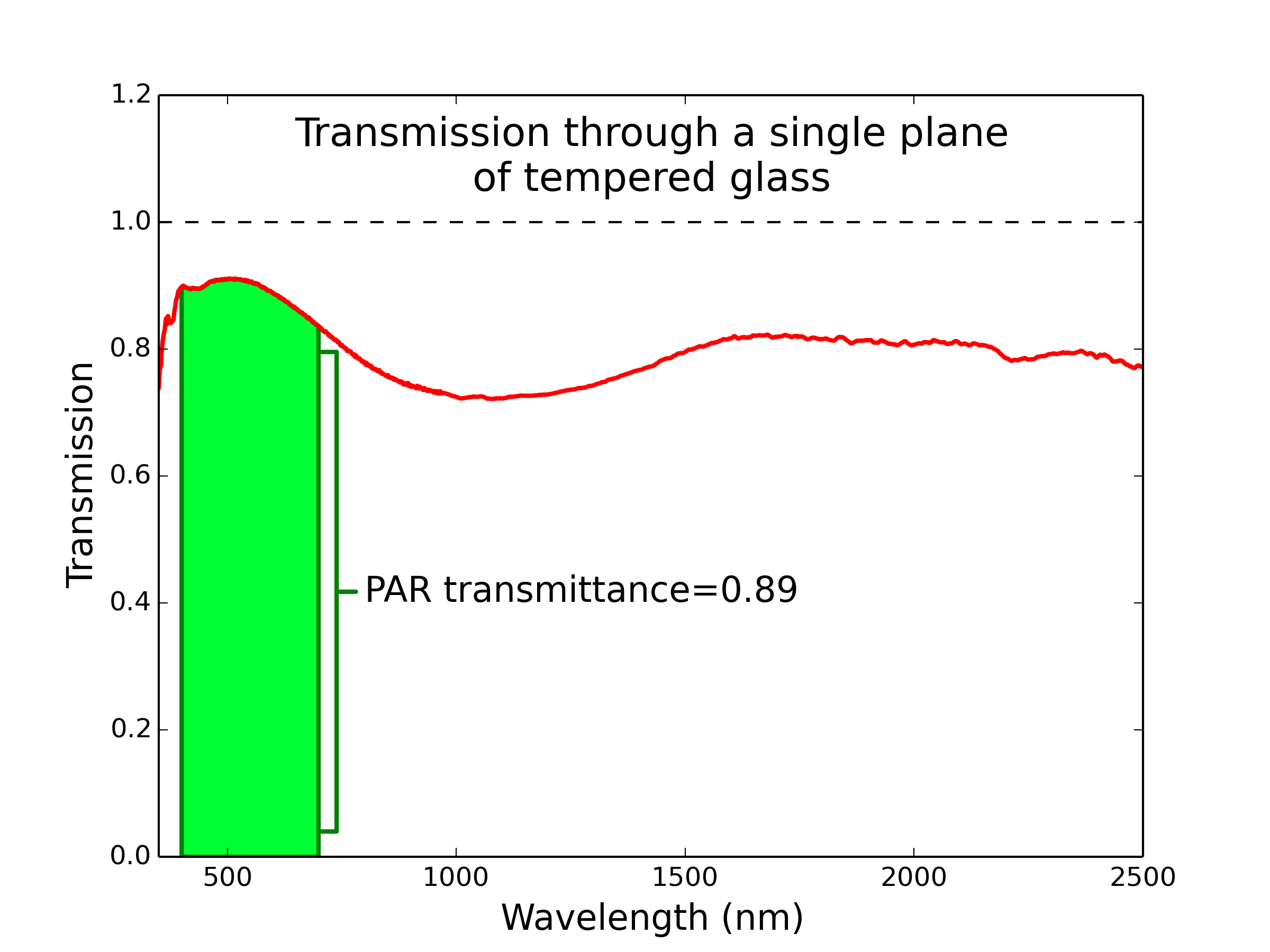

Supplement: S1 Fig — PAR was 89% transmitted. (TIF) [file pone.0138930.s001.tif]
