# Supplementary material for: Analysis of Environmental Effects on Leaf Temperature under Sunlight, High Pressure Sodium and Light Emitting Diodes
Source: PLoS One. 2015 Oct 8;10(10):e0138930. doi: 10.1371/journal.pone.0138930 (PMC4598144; doi:10.1371/journal.pone.0138930)
Supplement: S1 File — (PDF) [file pone.0138930.s002.pdf]

Supporting Information

Overview of code used to run the associated model.

```
#Variables
u = 1.5 #wind speed m/s
dmax = 10 #maximum length of leaf in cm
d = 0.72*(dmax/100) #characteristic dimension
hr = 0.5 #relative humidity
Rabs = 750 #radiation absorbed in W/m2
Tcel = 25 #air temperature is celcius
Tkel = Tcel+273.15
pa = 100 #air pressure in kPa
gvsab = 0.5 #stomatal conductace for vapor abaxial side, ranges from
    0.01 to 0.5
gvsad = 0.5 #stomatal conductace for vapor adaxial side, ranges from
    0.01 to 0.5
gvs=0.25

#Constants
lam = 44000 #lambda-the latent heat of evaporation J/molC
Cp = 29.3 #Cp-specific heat of air J/molC
a = 0.611 #SVP constant
b = 17.502 #SVP constant
c = 240.97 #SVP constant
sig = 5.67E-8 #sigma-Stephan Boltzman constant W/m2k4
eps = 0.97 #epsilon sub s-Emissivity of leaf
gamma = Cp/lam


#s-slope of saturation mole fraction function
def esfun(Tcel):
    a = 0.611 #SVP constant
    b = 17.502 #SVP constant
    c = 240.97 #SVP constant
    es = a*np.exp((b*Tcel)/(Tcel+c))
    return(es)

#slope of saturation mole fraction function: Tcel = air temp in celcius,
    pa = atmospheric pressure in kPa
def sfun(Tcel,pa):
    a = 0.611 #SVP constant
    b = 17.502 #SVP constant
    c = 240.97 #SVP constant
    es=esfun(Tcel)
    delta = (b*c*es)/(c+Tcel)**2
    return(delta/pa)
```

```
#gHr-boundry layer conductance for heat

def grfun(Tcel): #radiative conductance: Tcel = air temp in celcius
    Tkel = Tcel+273.15
    gr = (4*eps*sig*Tkel**3)/Cp
    return(gr)

def gHafun(u,d): #boundry layer conductance for heat: u = wind speed, d
     = characteristic dimension
    gHa = 0.135*np.sqrt(u/d)
    return(gHa)

gHr = grfun(Tcel)+gHafun(u,d)

##gv-conductance for vapor
def  gvafun(u,d): #boundry layer conductance for vapor: u = wind speed,
     d = characteristic dimension
    gva = 0.147*np.sqrt(u/d)
    return(gva)

def gvfun(gvsab,gvsad,gva): #conductance for vapor
    gvab = (0.5*gvsab*gva)/(gvsab+gva) #conductance of vapor from
        abaxial side
    gvad = (0.5*gvsad*gva)/(gvsad+gva) #conductance of vapor from
        adaxial side
    gv = gvab+gvad
    return(gv)


def TLitt(Rabs,Tcel,gvs,u,d,hr,pa):
    ea=esfun(Tcel)*hr
    Rabs=Rabs
    Tcel=Tcel
    gvs=gvs
    u=u
    d=d
    hr=hr
    pa=pa

    def TLinfun(x):
        #constants
        lam = 44000 #lambda-the latent heat of evaporation J/molC
        Cp = 29.3 #Cp-specific heat of air J/molC
        sig = 5.67E-8 #sigma-Stephan Boltzman constant W/m2k4
        eps = 0.97 #epsilon sub s-Emissivity of leaf

        #functions
        gHa = 1.4*gHafun(u,d)
        gva = 1.4*gvafun(u,d)
        gv = (gvs*gva)/(gvs+gva)
```

```
        xkel=x+273.15

        return(Rabs-eps*sig*(xkel**4)-Cp*gHa*(x-Tcel)-lam*gv*((esfun(x)-ea)/pa))

    TLit = fsolve(TLinfun,TLfun(Rabs,Tcel,gvs,u,d,hr,pa))
    return(TLit)
```
